# Disseminated embolization of an inferior vena cava filter with penetration of the aorta

**DOI:** 10.1093/ehjci/jeae124

**Published:** 2024-05-11

**Authors:** Marcus Brugger, Jochen Gaa, Katharina Bergmann

**Affiliations:** Department of Cardiology, Klinikum rechts der Isar, Technical University of Munich, Ismaninger Straße 22, 81675 Munich, Germany; Department of Radiology, Klinikum rechts der Isar, Technical University of Munich, Ismaninger Straße 22, 81675 Munich, Germany; Department of Cardiology, Klinikum rechts der Isar, Technical University of Munich, Ismaninger Straße 22, 81675 Munich, Germany

**Figure:**
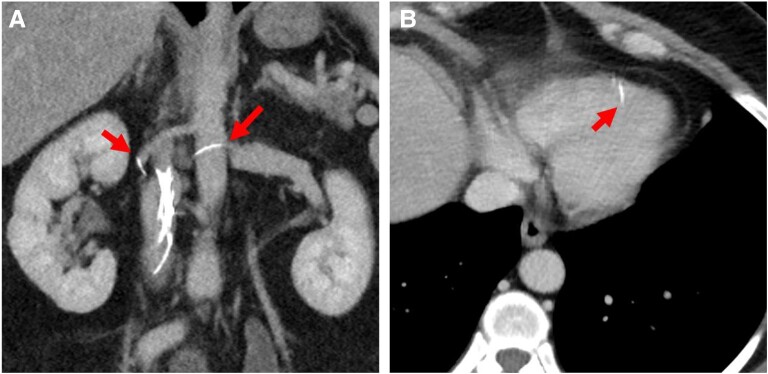


A 49-year-old patient was presented with compensated post-thrombotic syndrome in her left leg. In 2009, an inferior vena cava (IVC) filter was implanted due to iliofemoral deep vein thrombosis on the left side and bleeding while taking phenprocoumon as oral anticoagulation. To determine any thrombus burden in the area of the IVC filter, a CT angiography was performed at the MVZ Arabellahaus in Munich. This revealed a fractured IVC filter with penetration of the adjacent abdominal aorta and disseminated embolization of fragments into the left renal artery (*Panel A*), the pulmonary arteries, and the right ventricle (*Panel B*). The complication rate of a filter fracture is 1–2%, and the embolization of filter fragments is <1%. Penetration into neighbouring organs such as the intestine, aorta, or kidney occurs in 3.6% of cases and results in invasive therapy in 0.5%. In general, a cava filter that is no longer clinically indicated should be removed straightaway or implantation of temporary filters should be considered. Our patient was free of symptoms, so we refrained from any invasive therapy and planned clinical follow-ups.

**Funding:** None declared.

**Data availability:** No new data were generated or analysed in support of this research.

